# The Diagnostic Value of Transthoracic Echocardiography Parameters Under the New Diagnostic Criteria for Pulmonary Hypertension

**DOI:** 10.1155/carj/2592204

**Published:** 2025-10-23

**Authors:** Yuankun Qi, Junjun Liu, Xiaopei Cui, Yumiao Wang, Mingyuan Ma, Hongyu Zhang, Weida Lu, Min Xiang, Qiushang Ji

**Affiliations:** ^1^Key Laboratory of Ultra-Weak Magnetic Field Measurement Technology, School of Instrumentation and Optoelectronic Engineering, Beihang University, Ministry of Education, Beijing, China; ^2^Hangzhou Innovation Institute, Beihang University, Hangzhou, Zhejiang, China; ^3^Shandong Key Laboratory for Magnetic Field-free Medicine & Functional Imaging, Institute of Magnetic Field-free Medicine & Functional Imaging, Shandong University, Jinan, Shandong, China; ^4^Department of Geriatric Medicine, Qilu Hospital of Shandong University, Jinan, Shandong, China; ^5^Key Laboratory of Cardiovascular Proteomics of Shandong Province, Qilu Hospital of Shandong University, Jinan, Shandong, China; ^6^Jinan Clinical Research Center for Geriatric Medicine, Qilu Hospital of Shandong University, Jinan, Shandong, China; ^7^Department of Cardiology, Qilu Hospital of Shandong University, Jinan, Shandong, China

**Keywords:** diagnostic value, echocardiography, optimal threshold values, pulmonary hypertension, right heart catheterization

## Abstract

**Background:**

In 2022, new guidelines for the diagnosis and treatment of pulmonary hypertension (PH) revised the hemodynamic definition, reducing the mean pulmonary artery pressure threshold from ≥ 25 to > 20 mmHg. The optimal threshold of transthoracic echocardiography (TTE) parameters and the predictive capability require further validation. This study aims to investigate the diagnostic value of TTE parameters under the new hemodynamic criteria.

**Methods:**

Retrospective analysis of PH patients who underwent right heart catheterization and TTE examination between 2017 and 2022 in a single center. Logistic regression was employed to ascertain the predictive capacity of parameters across various conditions. Receiver operating characteristic curves were used to determine the optimal cutoff values based on the new criteria.

**Results:**

In a cohort of 213 patients, the optimal cutoff values identified were a tricuspid annular plane systolic excursion (TAPSE) to systolic pulmonary arterial pressure (sPAP) ratio of < 0.50 mm/mmHg, a right ventricular outflow tract acceleration time (RVOT-AT) of < 93 ms, and a right atrial area (RAA) > of 14.5 cm^2^. Regardless of the inclusion of tricuspid regurgitation velocity (TRV) and related parameters, RVOT-AT < 93 ms manifested as an effective predictive parameter. A combination of RVOT-AT < 93 ms, main pulmonary artery diameter > 25 mm and RAA > 14.5 cm^2^ exhibited better specificity.

**Conclusion:**

The threshold values for TAPSE/sPAP, RVOT-AT, and RAA should be adjusted to improve the predictive capacity of PH based on revised criteria in this single-center dataset. RVOT-AT was a promising indirect parameter, and the utilization of combined indirect indicators may enhance diagnostic accuracy, particularly in instances where satisfactory TRV measurements are unavailable.

## 1. Introduction

For nearly five decades, the hemodynamic diagnostic criteria for pulmonary hypertension (PH) have been defined as a mean pulmonary artery pressure (mPAP) of ≥ 25 mmHg, measured by right heart catheterization (RHC) at rest [1]. With the continuous accumulation of data on mPAP and pulmonary vascular resistance (PVR) in healthy individuals, the upper limit of normal range has gradually been clarified [2–4]. Moreover, recent studies indicated that even a mildly elevated mPAP adversely affects prognosis. A lower mPAP threshold may lead to earlier initiation of targeted therapy [3, 5–7]. Taking into account the aforementioned reasons and considerations, the 2022 European Society of Cardiology (ESC) and European Respiratory Society (ERS) guidelines for the diagnosis and treatment of PH have revised the diagnostic criterion for mPAP from ≥ 25 to > 20 mmHg [8].

Given the numerous complications and technical challenges associated with RHC, transthoracic echocardiography (TTE) has been validated as a highly effective alternative tool [9]. The guidelines emphasized that predicting PH using TTE cannot rely solely on the estimated systolic pulmonary arterial pressure (sPAP) derived from tricuspid regurgitation velocity (TRV) and estimated right atrial pressure (RAP). A comprehensive evaluation incorporating indirect indicators such as left and right heart morphology, left ventricular eccentricity index (LVEI), end-expiratory inferior vena cava diameter (IVC_E_), tricuspid annular plane systolic excursion (TAPSE), and right ventricular outflow tract acceleration time (RVOT-AT) is necessary [1]. Despite insufficient data to support the modification of cutoff values for relevant parameters of TTE, lowering the mPAP threshold will undeniably impact the accuracy of PH prediction by TTE. Previous study has demonstrated that the correlation between tricuspid regurgitation gradient (TRG) and sPAP measured by RHC is limited at low pressure levels [10]. However, the diagnostic performance of other TTE parameters is unknown under the new criteria. Therefore, further investigation is required to determine the optimal cutoff values and diagnostic performance of TTE parameters for predicting PH according to the revised diagnostic criteria.

The aim of this study was to determine the optimal cutoff values and diagnostic capability of various TTE parameters in patients with PH in accordance with the new hemodynamic diagnostic criteria.

## 2. Methods

### 2.1. Study Design and Population

A retrospective review was conducted on patients who underwent RHC at Qilu Hospital of Shandong University between July 2017 and December 2022. PH patients were classified into four groups based on their mPAP levels: patients with mPAP ≤ 20 mmHg were categorized as the non-PH group; those with mPAP between 21 and 24 mmHg were classified as PH according to the new diagnostic criteria; and those with mPAP ≥ 25 mmHg were diagnosed using traditional hemodynamic criteria. Furthermore, as stated in the 2015 ESC/ERS guidelines for the diagnosis and treatment of PH, in patients with PH associated with pulmonary disease, an mPAP ≥ 35 mmHg is classified as severe PH [1]. Therefore, we further subdivided the mPAP ≥ 25 mmHg group into two subgroups: the 25–34 mmHg group and the ≥ 35 mmHg group. All patients with a mPAP measured by RHC of ≤ 20 mmHg, between 21 and 24 mmHg, and with mPAP = 25–34 mmHg were selected. Given that the group with mPAP ≥ 35 mmHg included a relatively large number of patients, it was necessary to ensure balanced sample sizes across groups. Therefore, patients with mPAP ≥ 35 mmHg were selected and matched at a ratio of 1:2 to those with mPAP ≤ 20 mmHg, based on age and sex. A comparative analysis was conducted across the four groups to assess the influence of different levels of mPAP elevation on the TTE parameters. Meanwhile, the comparison between patients with mPAP ≤ 20 mmHg and the remaining three groups was performed to examine the diagnostic performance of TTE parameters under the newly established hemodynamic criteria. Patients with uncorrected congenital heart disease, right ventricular outflow tract stenosis, persistent atrial fibrillation, and diseases affecting the structure of the right heart were excluded. This study complied with the principles of the Declaration of Helsinki and was approved by the Ethics Committee of Shandong University of Qilu Hospital (No. KYLL-202210-054). Given that this is a retrospective study, both TTE and RHC are routine clinical examinations, and there is no need to obtain written informed consent from participants.

### 2.2. RHC

RHC was performed with a 131F7 Swan-Ganz catheter (Edwards, Lifesciences, Irvine, CA, USA) to obtain the hemodynamic parameters following standard procedure. Parameters measured included RAP, sPAP and mPAP, PVR, cardiac output (CO), cardiac index (CI), and mixed venous oxygen saturation (SvO_2_). CO and PVR were calculated following the standard formulas [11].

### 2.3. TTE

All patients underwent TTE examination as a part of clinical routine performed by a single experienced physician utilizing a GE Vivid E95 echocardiography machine (GE, Vingmed Ultrasound, Horten, Norway) within 24 h prior to RHC. TTE parameters included right ventricular diameter (RVD), left ventricular diameter (LVD), the ratio of RVD to LVD (RVD/LVD), right atrial area (RAA), main pulmonary artery diameter (mPA), the ratio of mPA to aorta (mPA/AO), the ratio of TAPSE to sPAP (TAPSE/sPAP), LVEI, IVC, RAP, sPAP, TRV, and RVOT-AT [12–14]. The estimation of RAP by TTE is based on the IVC_E_ and inferior vena cava collapsibility index (IVC_CI_) during inspiration and expiration [15]. When IVC_E_ > 21 mm and the IVC_CI_ < 50%, a high RAP level of 15 mmHg (range from 10 to 20 mmHg) is suggested. Conversely, when IVC_E_ < 21 mm with the IVC_CI_ > 50%, a normal RAP status of 3 mmHg (range from 0 to 5 mmHg) is suggested. When there is a unilateral abnormality in IVC_E_ and IVC_CI_, the estimated RAP is 8 mmHg (range 5–10 mmHg) [1].

To assess interobserver variability, the medical records of the patients were retrospectively analyzed. Specifically, patients who underwent TTE examinations performed by sonographers other than the “experienced physician” designated in this study within a 3-month period. Since the other sonographers were not specialized in right heart echocardiography, certain right heart functional parameters could not be reliably obtained (such as RVOT-AT and TAPSE). Therefore, only the following parameters were evaluated: LVD, RVD, RAA, mPA, TRV, sPAP, and RAP. To assess intraobserver variability, patients who underwent two TTE examinations performed by the same sonographer within 1 month, one being the routine TTE assessment before RHC and the other being the TTE examination during the outpatient follow-up.

### 2.4. Statistical Analysis

Statistical analyses were performed using the GraphPad Prism (v. 8.0.2, GraphPad Software, San Diego, CA, USA) and SPSS (Version 27, IBM Corp., Chicago, IL, USA). The normality of continuous data was assessed using the Shapiro–Wilk test. Continuous data adhering to a normal distribution were represented as the mean ± standard deviation, while non-normally distributed data were represented as the median (interquartile range). Categorical variables were expressed as counts and percentages. One-way ANOVA was utilized to compare means among multiple groups of parameters, while the Kruskal–Wallis H test was applied to compare the medians of variables across different groups. If there was a significant difference between groups, post hoc test was used to compare groups (Tukey HSD test for one-way ANOVA and Bonferroni-adjusted Dunn test for Kruskal–Wallis H test). Parameters that demonstrated statistical significance (*p* < 0.05) in the univariate logistic regression analysis were subsequently included in the multivariate logistic regression analysis, where forward selection based on likelihood ratio statistics was applied for variable screening. Pearson correlation analysis was performed to assess the correlation between two normally distributed parameters, while Spearman correlation analysis was employed for non-normally distributed data. The intraclass correlation coefficient (ICC) was computed, and the Bland–Altman method was used to analyze the agreement between two methods as well as intra- and interobserver variabilities. Receiver operating characteristic (ROC) curves were used to determine the optimal cutoff values based on the Youden Index according to the thresholds to diagnose PH defined by mPAP > 20 mmHg. DeLong's test was used to compare the difference in the area under the ROC curve (AUC). Sensitivity, specificity, positive predictive value (PPV), negative predictive value (NPV), and accuracy were calculated. For all tests, *p* < 0.05 was considered statistically significant.

## 3. Results

### 3.1. Patient Characteristics and Parameters of TTE and RHC

**Figure 1:**
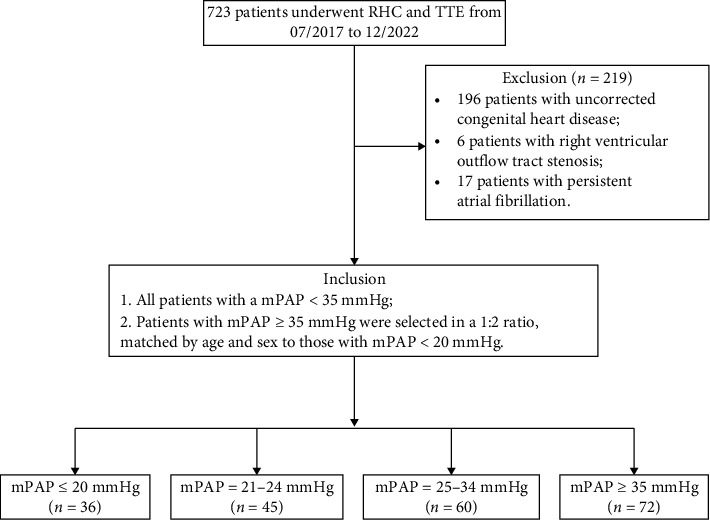
Flow chart of patients' screening and subgroups.

A total of 213 patients were included in this study. Among these patients, 175 were female (82.2%), with a mean age of 43.3 ± 16.1 years. Patients were categorized into four groups based on mPAP measured by RHC. The study included 36 patients with mPAP ≤ 20 mmHg, 45 patients with mPAP ranging from 21 to 24 mmHg, 60 patients with mPAP ranging from 25 to 34 mmHg, and 72 patients with mPAP ≥ 35 mmHg (Figure 1). The RVOT-AT, mPA, sPAP, TAPSE/sPAP, and TRV of TTE demonstrated significant differences between patients with mPAP = 21–24 mmHg and patients with mPAP = 25–34 mmHg compared to patients with mPAP ≤ 20 mmHg (Table 1). Comparing patients with mPAP = 21–24 mmHg and mPAP = 25–34 mmHg revealed no statistically significant differences in most parameters, except for sPAP, TAPSE/sPAP, and TRV. Regarding RHC parameters, no statistical differences were observed in CO and CI among the four groups (*p* = 0.093 and 0.074, respectively). The RAP of patients with mPAP ≥ 35 mmHg was higher compared to either patients with mPAP ≤ 20 mmHg or mPAP = 25–34 mmHg. Additionally, the demographic characteristics and specific medications between different groups are presented in Table S1.

### 3.2. Optimal Cutoff Values of TTE Parameters and Diagnostic Efficacy for PH Based on the New Criteria

The sensitivity, specificity, PPV, NPV, and accuracy of each parameter in predicting mPAP > 20 mmHg are summarized in Table 2. The results indicated that the NPV of all parameters for diagnosing mPAP > 20 mmHg was limited to less than 50%, with the exception of RVOT-AT (< 93 ms) and TRV (> 2.8 m/s). Among these parameters, TRV (> 2.8 m/s), RVOT-AT (< 93 ms), and sPAP (> 37.5 mmHg) exhibited notable sensitivity at 90.0%, 88.6%, and 84.0%, respectively. Compared to other parameters, LVEI (> 1.1) achieved the highest specificity at 93.9%, though its sensitivity was only 42.3%. Additionally, TAPSE/sPAP (< 0.50 mm/mmHg) demonstrated a high specificity of 80.0%. The TRV (> 2.8 m/s), RVOT-AT (< 93 ms), and sPAP (> 37.5 mmHg) were with higher accuracy at 84.7%, 83.9%, and 82.2%, respectively. It was found that the optimal threshold values of TAPSE/sPAP, RVOT-AT, and RAA deviated from the new guidelines. Compared to the cutoff values recommended by the guidelines, while TAPSE/sPAP (< 0.50 mm/mmHg) and RVOT-AT (< 93 ms) showed a slight reduction in sensitivity, they substantially enhanced both specificity and PPV. For RAA (> 14.5 cm^2^), this adjustment significantly improved sensitivity.

### 3.3. Predictive Value of TTE Parameters

Univariate logistic regression analysis revealed that all TTE parameters exhibited significant predictive values (Table S2).

### 3.4. Predictive Value of TTE Parameters Based on Tricuspid Regurgitation (TR) Condition

**Figure 2:**
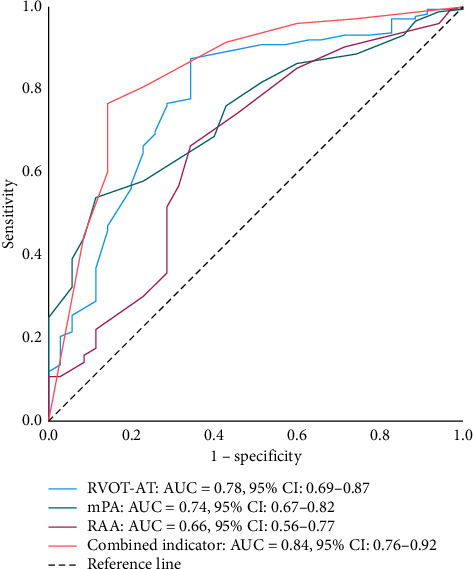
Receiver operating characteristic curves of combined indicators. Red line presented the combined indicator. mPA, main pulmonary artery diameter; RAA, right atrial area; RVOT-AT, right ventricular outflow tract acceleration time.

When TR was minimal or absent, a multivariate logistic regression analysis was performed using parameters that excluded TRV, sPAP, and TAPSE/sPAP. The covariates incorporated into the logistic regression model were RVOT-AT (< 93 ms), mPA (> 25 mm), mPA/AO (> 1), LVEI (> 1.1), RAA (> 14.5 cm^2^), RVD (> 26.5 mm), RVD/LVD (> 0.65), and IVC_E_ (> 13.5 mm). The results demonstrated that the RVOT-AT < 93 ms (OR 10.68, 95% CI 4.16–27.45, *p* < 0.001), mPA > 25 mm (OR 4.13, 95% CI 1.59–10.74, *p*=0.004), and RAA > 14.5 cm^2^ (OR 3.23, 95% CI 1.22–8.56, *p*=0.018) exhibited significant predictive value (Table 3). ROC curves were constructed based on the RVOT-AT, mPA, RAA, and their combined indicator (Figure 2). The AUC for combined indicator was 0.84 (95% CI 0.76–0.92, *p* < 0.001), which had significant difference between mPA and RAA (0.84 vs. 0.74 and 0.66, respectively; *p*=0.024 and 0.001, respectively), but it was statistically equivalent to RVOT-AT (0.84 vs. 0.79, *p*=0.164). The sensitivity, specificity, PPV, NPV, and accuracy were 76.3%, 85.7%, 96.4%, 41.7%, and 77.8%, respectively, for combined indicator.

When the TR was available, covariates of the logistic regression were further expanded to include sPAP (> 37.5 mmHg), RVOT-AT (< 93 ms), and TAPSE/sPAP (< 0.50 mm/mmHg), building upon the previous step. The results showed that the RVOT-AT < 93 ms and sPAP > 37.5 mmHg presented predictive value (Table 3).

Building on the TR scenarios established under the two preceding assumptions, logistic regression with the Enter method was employed to simultaneously incorporate all candidate variables into the model. This approach enabled a comprehensive assessment of the independent contribution of each variable, while controlling for other covariates, thereby mitigating the risk of overlooking potential confounding factors inherent to stepwise selection methods. The results demonstrated that RVOT-AT (< 93 ms) retained statistical significance irrespective of the availability of TR (Table S3).

Drawing on the findings of different TR conditions, the recommended TTE parameters and their corresponding cutoff values are detailed in Table 4.

### 3.5. RAP Between Two Methods

**Figure 3:**
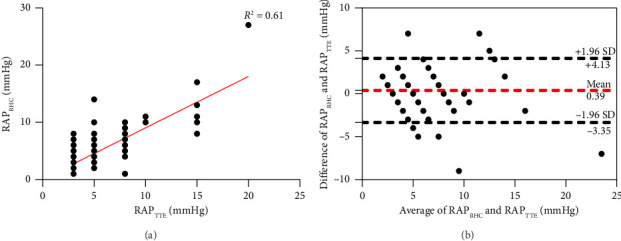
Relationship between right atrial pressure (RAP) measured by right heart catheterization (RAP_RHC_) and transthoracic echocardiography (RAP_TTE_). (a) Scatter plot of RAP_RHC_ and RAP_TTE_. The regression equation is RAP_RHC_ = 0.89 × RAP_TTE_ + 0.06, *R*^2^ = 0.61. (b) Bland–Altman analysis of two methods. The mean difference is 0.39 mmHg, and the black broken lines represent the 95% limits of agreement (4.13 to −3.35 mmHg).

Accurate estimation of RAP is essential to ensuring the precision of sPAP. Therefore, a systematic comparative analysis of RAP values obtained from TTE and RHC is warranted. The results demonstrated that RAP_TTE_ and RAP_RHC_ exhibited a moderate correlation across the entire cohort (*r* = 0.59, *p* < 0.001, Figure 3(a)). According to the Bland–Altman analysis, the bias of two methods was 0.39 mmHg, with wide 95% limits of agreement (Figure 3(b)).

### 3.6. Intraobserver and Interobserver Variability

The analysis revealed that interobserver reproducibility for TTE parameters was satisfactory, with ICCs consistently exceeding 0.70 and *p* < 0.05. Moreover, intraobserver reproducibility for the same parameters was excellent, with ICCs surpassing 0.80 (Table S4). The Bland–Altman analysis demonstrated nearly all differences in TTE parameters fell within the 95% limits of agreement for both intraobserver and interobserver variability (Figure S1).

## 4. Discussion

This single-center, retrospective study validates the optimal cutoff values for TTE parameters recommended in guidelines under the updated PH diagnostic standard. Our findings indicate that with standardized and precise RAP estimation, sPAP demonstrates superior efficacy over TRV in identifying PH based on the new diagnostic criteria. Furthermore, the cutoff values for certain TTE parameters warrant adjustment to align with the updated diagnostic criteria.

In the wake of the 6th World Symposium on PH, cardiologists have validated the optimal threshold values of TTE parameters for PH screening, utilizing mPAP > 20 mmHg as the benchmark. These investigations consistently identified TRV as the critical parameter in this context. Sumimoto et al. and D'Alto et al. both investigated the diagnostic capability of various TRV values for PH based on the new criteria, respectively [16, 17]. Their findings indicated that TRV > 2.8 m/s served as the optimal threshold value and performed very well in term of sensitivity and specificity. However, Mandoli et al. proposed a lower TRV cutoff of 2.4 m/s to enhance sensitivity (44% vs. 65%), albeit at the expense of specificity [18]. They asserted that the sensitivity should be prioritized over specificity in the screening of PH. In the present study, we also demonstrated that TRV > 2.8 m/s is sensitive in PH screening, albeit with a low specificity (57.6%), necessitating the investigation of a more robust index.

Estimates of sPAP are based on the TRG taking into account non-invasive estimates of RAP. The ESC/ERS guidelines recommend peak TRV, rather than estimated sPAP, as the primary variable for determining the echocardiographic probability of PH, considering the inaccuracies in estimating RAP and the amplification of measurement errors [19, 20].

RAP was estimated based on the IVC_E_ and IVC_CI_ during inspiration and expiration which may introduce significant errors in the sPAP measurements obtained by TTE. Numerous studies have investigated the correlation and consistency between RAP measurements obtained via TTE and RHC [21–23]. In our study, the relationship of RAP_TTE_ and RAP_RHC_ was consistent with previous findings, exhibiting a significant correlation and wide 95% limits of agreement. Our study demonstrated that following consistent and strict standards of RAP estimation, sPAP (> 37.5 mmHg) exhibited superior specificity compared to TRV (> 2.8 m/s) (72.7% vs. 57.6%), although the sensitivity was marginally lower (84% vs. 90%). This is challenging in widely use only if that echocardiographers undergo rigorous training in RAP estimation. We did not try to lower the TRV threshold here because Gall et al. [10] already indicated that compared to TRG of 31 mmHg (equivalent to TRV = 2.8 m/s), using a threshold of 26 mmHg for screening PH patients based on the new criteria may result in extremely low specificity (25%), despite a slight improvement in sensitivity (93% vs. 97%). We do agree that the current TRG or TRV threshold for screening PH should not be modified without substantial empirical evidence.

Patients with mPAP = 21–24 mmHg represent a newly included group based on the revised diagnostic criteria, and mPAP = 25–34 mmHg represents patients previously classified as mild PH. There were no significant differences in structural TTE parameters between these two groups. However, in comparison to the three groups, patients with mPAP ≥ 35 mmHg exhibited discernible alterations in cardiac morphology, thereby substantiating the pivotal role of elevated pulmonary artery pressure in the remodeling of the right heart [24].

TAPSE/sPAP more accurately reflects the load status of pulmonary circulation and serve as an independent prognostic TTE parameter [25, 26]. The 2022 ESC/ERS guidelines recommend TAPSE/sPAP < 0.55 mm/mmHg as a key indirect sign for predicting PH. However, our study revealed that although it exhibited a high sensitivity of 88.0%, its specificity was inadequate at only 60.0%. Compared to TAPSE/sPAP < 0.50 mm/mmHg, it did not represent a more optimal threshold. Although the sensitivity of TAPSE/sPAP < 0.50 mm/mmHg was slightly reduced (79.6% vs. 88.0%), its specificity was improved to 80.0%. Gall et al. recommended an even lower threshold (< 0.36 mm/mmHg) with high specificity but poor sensitivity [10]. Huston and colleagues retrospectively analyzed 47,728 patients who underwent TTE examination, revealing that the cutoff value for TAPSE/sPAP in mild PH was 0.55 mm/mmHg [27]. The guideline referenced this study as the basis for establishing the TAPSE/sPAP cutoff value. However, Huston's study enrolled patients with mild PH (sPAP_TTE_ = 33–39 mmHg), whereas our study included a broader spectrum of patients, ranging from mild to moderate and severe PH. Consequently, the difference is likely the primary factor contributing to the lower TAPSE/sPAP cutoff value observed in this study. Similarly, our study found that, apart from TAPSE/sPAP, the optimal cutoff values of RAA and RVOT-AT differed from those recommended by the guidelines. This discrepancy may be attributed to the composition of the study population. For example, the proportion of patients with postcapillary PH or combined postcapillary and precapillary PH was relatively low in our study. The interaction between the left and right heart might influence the ability of these parameters to distinguish PH [28].

Accurate estimation of TRV poses significant challenges, particularly in patients with severe or very little TR, misinterpretation of tricuspid valve closure artifact for the TR jet, or incorrect assignment of a peak TRV in the case of maximum velocity boundary artifacts [17, 29]. RVOT-AT reflects the afterload of the right ventricle, exhibiting a strong correlation with sPAP and remaining independent of TR severity [30–32]. Our observations indicated that the recommended cutoff value of RVOT-AT < 105 ms by the guideline may not be appropriate for PH screening according to new diagnostic criteria. Consequently, we sought to investigate whether a new cutoff value < 93 ms according to our data improved sensitivity and specificity. However, the specificity for RVOT-AT < 93 ms remained suboptimal. Therefore, we conducted logistic regression analysis, excluding sPAP in cases with insufficient TR signal quality, a common issue in clinical practice. The RVOT-AT < 93 ms remained its predictive value, alongside mPA > 25 mm and RAA > 14.5 cm^2^. Combination of these three parameters did not superior to RVOT-AT (*p* > 0.05 in the DeLong test). Consequently, in instances where TR cannot be accurately assessed, RVOT-AT may serve as the primary diagnostic parameter. Meanwhile, mPA and RAA, which reflect cardiac remodeling and the consequences of PH, can function as secondary parameters within the predictive model, thereby enhancing diagnostic accuracy when TR quality is suboptimal. According to our findings, although diagnostic performance of mPA and RAA was relatively suboptimal, their integration with RVOT-AT to construct a predictive model led to an improvement in diagnostic specificity compared to the use of RVOT-AT alone. In the presence of a TR signal, although TAPSE/sPAP was excluded from the logistic regression model, its exceptional sensitivity and specificity justify its inclusion among the indirect TTE parameters for PH evaluation.

There are several limitations to our study. Firstly, this is a single-center, retrospective study with sample size bias being an unavoidable factor. Due to sample size limitations, we were unable to stratify the various PH phenotypes, thus hindering the determination of optimal cutoff values for each TTE parameter across different PH classifications. Secondly, the lack of external validation may cast doubt on the robustness of the results. Next, we should conduct multicenter prospective studies, which are particularly important for TTE. Meanwhile, by expanding the sample size and recruiting patients with different classifications of PH, we can further refine and extend the findings of this study. Finally, the relatively low number of negative patients (mPAP < 20 mmHg) led to an imbalance between groups, which in turn affected the NPV. Additionally, previous studies have demonstrated that both arrhythmias and diuretic therapy can influence the measurements of sPAP and RVOT-AT [33, 34]. Apart from excluding patients with persistent atrial fibrillation, our study did not further stratify due to the inability to ascertain whether patients had been administered diuretics or had other forms of arrhythmias at the time of the TTE examination.

## 5. Conclusion

In this single-center study, based on the revised diagnostic criteria for PH, TAPSE/sPAP < 0.50 mm/mmHg, RVOT-AT < 93 ms, and RAA > 14.5 cm^2^ appear to perform better in identifying PH than the threshold values currently suggested by guidelines. In this small dataset, sPAP performed better as a direct parameter compared to TRV, while RVOT-AT was a promising indirect parameter. When TRV is unavailable or unreliable, the combined use of multiple indirect parameters can enhance predictive performance. Given that this study was based on a relatively small single-center sample, the results still need to be further validated by large-scale, multicenter studies.

## Figures and Tables

**Table 1:** RHC and TTE parameters among different groups.

Parameters	mPAP ≤ 20 mmHg (*n* = 36)	mPAP = 21–24 mmHg (*n* = 45)	mPAP = 25–34 mmHg (*n* = 60)	mPAP ≥ 35 mmHg (*n* = 72)	*p*
*RHC parameters*					
sPAP (mmHg)	26.83 ± 4.86	34.07 ± 4.45^※^	46.32 ± 7.70^※▀^	80.21 ± 23.85^※▀▲^	< 0.001
mPAP (mmHg)	15.83 ± 3.08	22.38 ± 1.03^※^	29.33 ± 2.70^※▀^	49.18 ± 14.84^※▀▲^	< 0.001
PVR (WU)	1.75 (1.05, 2.10)	2.91 ± 0.96^※^	4.28 (3.36, 5.46)^※▀^	8.04 (5.80, 11.83)^※▀▲^	< 0.001
CO (L/min)	5.56 ± 1.18	5.42 ± 1.58	5.12 ± 1.32	4.90 ± 1.46^※^	0.093
CI (L/min·m^2^)	3.38 ± 0.75	3.37 ± 0.95	3.21 ± 0.76	3.00 ± 0.86^※▀^	0.074
RAP (mmHg)	3.00 (2.00, 4.00)	4.00 (2.00, 5.00)	3.00 (2.00, 4.00)	4.00 (3.00, 6.00)^※▲^	0.001
PAWP (mmHg)	8.00 (5.00, 9.00)	6.00 (5.00, 9.00)	6.00 (5.00, 9.00)	6.21 ± 2.73^※^	0.103

*TTE parameters*					
RVOT-AT (ms)	98.37 ± 23.03	82.22 ± 21.01^※^	81.72 ± 17.26^※^	68.24 ± 15.28^※▀▲^	< 0.001
mPA (mm)	23.91 ± 3.42	27.40 ± 5.42^※^	27.12 ± 6.14^※^	31.33 ± 7.01^※▀▲^	< 0.001
LVEI	0.98 (0.90, 0.99)	0.98 (0.89, 1.01)	0.98 (0.97, 1.04)	1.23 (1.12, 1.66)^※▀▲^	< 0.001
TAPSE (mm)	19.94 ± 3.50	19.68 ± 3.10	18.74 ± 4.27	17.28 ± 3.73^※▀▲^	0.001
sPAP (mmHg)	34.24 ± 6.69	41.07 ± 7.21^※^	47.15 ± 12.52^※▀^	75.49 ± 21.66^※▀▲^	< 0.001
TAPSE/sPAP (mm/mmHg)	0.61 ± 0.16	0.49 ± 0.10^※^	0.43 ± 0.16^※▀^	0.25 ± 0.10^※▀▲^	< 0.001
TRV (m/s)	2.75 ± 0.29	3.02 ± 0.28^※^	3.26 ± 0.45^※▀^	4.13 ± 0.63^※▀▲^	< 0.001
RVD (mm)	24.44 ± 5.50	26.67 ± 6.65	26.19 ± 4.55	35.76 ± 8.97^※▀▲^	< 0.001
RVD/LVD	0.56 (0.51, 0.64)	0.59 (0.55, 0.67)	0.61 (0.53, 0.72)	0.97 (0.71, 1.42)^※▀▲^	< 0.001
mPA/AO	0.97 ± 0.18	1.05 ± 0.25	1.07 ± 0.29	1.27 ± 0.30^※▀▲^	< 0.001
RAP (mmHg)	3.00 (3.00, 4.50)	3.00 (3.00, 5.00)	3.00 (3.00, 5.00)	5.00 (3.00, 8.00)^※▲^	< 0.001
RAA (cm^2^)	13.00 (11.00,19.00)	14.00 (13.00, 18.50)	15.00 (13.00, 17.00)	20.00 (15.00, 26.75)^※▀▲^	< 0.001
IVC_E_ (mm)	12.88 ± 3.13	13.78 ± 3.86	14.57 ± 3.44^※^	17.43 ± 4.87^※▀▲^	< 0.001

*Note:* AO, aorta diameter; IVC_E_, inferior vena cava diameter of end-expiratory; mPA, main pulmonary artery diameter; TTE, transthoracic echocardiography.

Abbreviations: CI, cardiac index; CO, cardiac output; LVD, left ventricular diameter; LVEI, left ventricular eccentricity index; mPAP, mean pulmonary artery pressure; PVR, pulmonary vascular resistance; RAA, right atrial area; RAP, right atrial pressure; RHC, right heart catheterization; RVD, right ventricular diameter; RVOT-AT, right ventricular outflow tract acceleration time; sPAP, systolic pulmonary arterial pressure; TAPSE, tricuspid annular plane systolic excursion; TRV, tricuspid regurgitation velocity.

^※^ *p* < 0.05 vs. patients with mPAP ≤ 20 mmHg.

^▀^ *p* < 0.05 vs. patients with mPAP = 21–24 mmHg.

^▲^ *p* < 0.05 vs. patients with mPAP = 25–34 mmHg.

**Table 2:** The optimal cutoff values of TTE parameters and the diagnostic efficacy for new criterion.

Parameters	Cutoff values	Sen (%)	Spe (%)	PPV (%)	NPV (%)	Acc (%)	*p*
TAPSE/sPAP	< 0.50 mm/mmHg	79.6	80.0	95.7	41.4	79.7	< 0.001
TAPSE/sPAP	< 0.55 mm/mmHg^※^	88.0	60.0	92.5	47.4	83.8	< 0.001
RVOT-AT	< 93 ms	88.6	60.0	91.8	51.2	83.9	< 0.001
RVOT-AT	< 105 ms^※^	92.0	34.3	87.6	46.2	82.5	< 0.001
RAA	> 14.5 cm^2^	66.9	69.4	91.4	30.1	67.3	< 0.001
RAA	> 18 cm^2※^	35.8	72.2	86.3	18.7	42.0	0.465
mPA	> 25 mm	68.8	60.0	89.6	27.6	67.1	0.002
LVEI	> 1.1	42.3	93.9	97.3	24.2	50.7	< 0.001
sPAP	> 37.5 mmHg	84.0	72.7	94.0	47.1	82.2	< 0.001
TRV	> 2.8 m/s	90.0	57.6	91.6	52.8	84.7	< 0.001
RVD	> 26.5 mm	60.6	77.8	93.0	28.9	63.5	< 0.001
RVD/LVD	> 0.65	58.0	77.8	92.7	27.5	61.3	< 0.001
mPA/AO	> 1	60.0	68.6	90.5	25.5	61.4	0.003
IVC_E_	> 13.5 mm	63.1	68.8	91.7	25.3	63.9	0.002

*Note:* Acc, accuracy; AO, aorta diameter; IVC_E_, inferior vena cava diameter of end-expiratory; mPA, main pulmonary artery diameter; Sen, sensitivity; Spe, specificity; TTE, transthoracic echocardiography.

Abbreviations: LVD, left ventricular diameter; LVEI, left ventricular eccentricity index; NPV, negative predictive value; PPV, positive predictive value; RAA, right atrial area; RVD, right ventricular diameter; RVOT-AT, right ventricular outflow tract acceleration time; sPAP, systolic pulmonary arterial pressure; TAPSE, tricuspid annular plane systolic excursion; TRV, tricuspid regurgitation velocity.

^※^The cutoff values recommended by guidelines.

**Table 3:** Multivariate logistic regression analysis for identifying parameters with predictive value for PH.

Parameters^※^	Odds ratio	95% CI	*p*
*Excluding TR-related parameters*			
RVOT-AT (< 93 ms)	10.68	4.16–27.45	< 0.001
mPA (> 25 mm)	4.13	1.59–10.74	0.004
RAA (> 14.5 cm^2^)	3.23	1.22–8.56	0.018

*Indexing sPAP and TRV*			
RVOT-AT (< 93 ms)	6.98	2.58–18.85	< 0.001
sPAP (> 37.5 mmHg)	10.12	3.68–27.86	< 0.001

*Note:* mPA, main pulmonary artery diameter.

Abbreviations: CI, confidence interval; PH, pulmonary hypertension; RAA, right atrial area; RVOT-AT, right ventricular outflow tract acceleration time; sPAP, systolic pulmonary arterial pressure; TRV, tricuspid regurgitation velocity.

^※^Parameters were assessed with a logistic regression model with a forward stepwise likelihood ratio.

**Table 4:** Recommended TTE parameters and corresponding cutoff values for the diagnosis of PH on the new criterion.

Parameters	Cutoff values
*TR signal is mild or unavailable*	
RVOT-AT	< 93 ms
mPA	> 25 mm
RAA	> 14.5 cm^2^

*TR signal is clear and available*	
TAPSE/sPAP	< 0.50 mm/mmHg
RVOT-AT	< 93 ms
sPAP	> 37.5 mmHg

*Note:* mPA, main pulmonary artery diameter.

Abbreviations: RAA, right atrial area; RVOT-AT, right ventricular outflow tract acceleration time; sPAP, systolic pulmonary arterial pressure; TAPSE, tricuspid annular plane systolic excursion; TR, tricuspid regurgitation.

## Data Availability

The data that support the findings of this study are available from the corresponding author (Qiushang Ji, E-mail: jiqius@aliyun.com) upon reasonable request.

## Supporting Information

Supporting Table 1: Demographic Characteristics and Specific Medications between Different Groups.

Supporting Table 2: Univariate logistic regression analysis for identifying parameters with predictive values for PH.

Supporting Table 3: Multivariate logistic regression (Enter method) including all parameters across different TR scenarios.

Supporting Table 4: The inter- and intraobserver variability of certain TTE parameters determined by interclass correlation coefficients.

Supporting Figure 1: The Bland–Altman plots present intraobserver and interobserver variabilities of TTE parameters.
