# Correction to “The Diagnostic Value of Transthoracic Echocardiography Parameters Under the New Diagnostic Criteria for Pulmonary Hypertension”

**DOI:** 10.1155/carj/9809063

**Published:** 2026-10-28

Y. Qi, J. Liu, X. Cui, et al., “The Diagnostic Value of Transthoracic Echocardiography Parameters Under the New Diagnostic Criteria for Pulmonary Hypertension,” *Canadian Respiratory Journal* 2025 (2025): 2592204, https://doi.org/10.1155/carj/2592204.

In the article titled “The Diagnostic Value of Transthoracic Echocardiography Parameters Under the New Diagnostic Criteria for Pulmonary Hypertension,” the Author Contributions section, the statement “Yuankun Qi and Junjun Liu have contributed equally to this work.” was included in error. This statement should be removed.

We apologize for this error.

