# Supplementary material for: The Diagnostic Value of Transthoracic Echocardiography Parameters Under the New Diagnostic Criteria for Pulmonary Hypertension
Source: Can Respir J. 2025 Oct 23;2025:2592204. doi: 10.1155/carj/2592204 (PMC12575021; doi:10.1155/carj/2592204)
Supplement: Supporting Information — Additional supporting information can be found online in the Supporting Information section. [file 2592204.f1.docx]

**Supporting information:**

**Table S1**. Demographic Characteristics and Specific Medications between Different Groups.

| Characteristics | mPAP ≤ 20  mmHg (n = 36) | mPAP = 21-24 mmHg (n = 45) | mPAP = 25-34  mmHg (n = 60) | mPAP ≥ 35  mmHg (n = 72) |
| --- | --- | --- | --- | --- |
| Female | 29 (80.6%) | 40 (88.9%) | 50 (83.3%) | 56 (77.8%) |
| Age | 44.14 ± 17.94 | 43.82 ± 15.28 | 45.18 ± 14.33 | 41.64 ± 18.31 |
| BMI | 24.05 ± 4.88 | 23.16 ± 4.02 | 22.99 ± 3.37 | 23.40 ± 3.38 |
| Classification |  |  |  |  |
| IPAH | N/A | 7 (15.6%) | 8 (13.3%) | 29 (40.3%) |
| CHD | N/A | 16 (35.6%) | 15 (25.0%) | 10 (13.9%) |
| CTD | N/A | 10 (22.2%) | 16 (26.7%) | 4 (5.6%) |
| HPAH | N/A | 0 | 1 (1.7%) | 4 (5.6%) |
| PoPH | N/A | 1 (2.2%) | 2 (3.3%) | 4 (5.6%) |
| LD | N/A | 3 (6.7%) | 2 (3.3%) | 3 (4.2%) |
| LHD | N/A | 2 (4.4%) | 2 (3.3%) | 1 (1.4%) |
| CTEPH | N/A | 6 (13.3%) | 14 (23.3%) | 17 (23.6%) |
| Received PAH therapy | N/A | n = 17 | n = 53 | n = 71 |
| Therapy type^†^ |  |  |  |  |
| Single^*^ |  |  |  |  |
| PDE5I | N/A | 1 (5.9%) | 10 (18.9%) | 8 (11.3%) |
| ERA | N/A | 11 (64.7%) | 12 (23.1%) | 5 (7.0%) |
| sGCS | N/A | 0 | 3 (5.7%) | 3 (4.2%) |
| PA | N/A | 0 | 3 (5.7%) | 0 |
| CCB | N/A | 0 | 0 | 1 (1.4%) |
| Dual^⁑^ |  |  |  |  |
| ERA+PDE5I | N/A | 2 (11.8%) | 24 (45.3%) | 21 (29.6%) |
| ERA+sGCS | N/A | 0 | 0 | 2 (2.8%) |
| ERA+CCB | N/A | 0 | 0 | 1 (1.4%) |
| PDE5I+PCA | N/A | 0 | 0 | 2 (2.8%) |
| Triple^⁂^ |  |  |  |  |
| PCA+ ERA+PDE5I | N/A | 3 (17.6%) | 1 (1.9%) | 18 (25.4%) |
| PCA+ ERA+sGCS | N/A | 0 | 0 | 2 (2.8%) |
| ERA+PDE5I+PA | N/A | 0 | 0 | 7 (9.9%) |
| ERA+PDE5I+CCB | N/A | 0 | 0 | 1 (1.4%) |

BMI, body mass index; CCB, calcium channel blockers; CHD, congenital heart disease; CTD, connective tissue disease; CTEPH, chronic thromboembolic pulmonary hypertension; ERA, endothelin receptor antagonists; HPAH, heritable pulmonary arterial hypertension; IPAH, idiopathic pulmonary arterial hypertension; LD, pulmonary hypertension associated with lung diseases; LHD, pulmonary hypertension associated with left heart diseases; PA, prostacyclin analogues; PCA, prostacyclin receptor agonist; PDE5I, phosphodiesterase 5 inhibitors; PoPH, portopulmonary hypertension; sGCS, soluble guanylate cyclase stimulator.

^†^Among those who received PAH therapy; * Among those who received single therapy; ⁑ Among those who received dual therapy; ⁂ Among those who received triple therapy

**Table S2** Univariate logistic regression analysis for identifying parameters with predictive values for PH.

| Parameters | Odds Ratio | 95% CI | *p* |
| --- | --- | --- | --- |
| TAPSE/sPAP (<0.50 mm/mmHg) | 15.77 | 5.97-41.61 | <0.001 |
| RVOT-AT (<93 ms) | 11.78 | 5.18-26.76 | <0.001 |
| mPA (>25 mm) | 3.30 | 1.56-6.97 | 0.002 |
| LVEI (> 1.1) | 11.51 | 2.67-49.64 | 0.001 |
| sPAP (> 37.5 mmHg) | 14.12 | 5.92-33.70 | <0.001 |
| TRV (> 2.8 m/s) | 12.21 | 5.21-28.66 | <0.001 |
| RVD (> 26.5 mm) | 5.30 | 2.28-12.30 | <0.001 |
| RVD/LVD (> 0.65) | 4.76 | 2.05-11.03 | <0.001 |
| mPA/AO (> 1) | 3.30 | 1.52-7.17 | 0.003 |
| RAA (> 14.5cm^2^) | 4.51 | 2.08-9.78 | <0.001 |
| IVC_E_ (> 13.5 mm) | 3.79 | 1.69-8.50 | 0.001 |

AO, aorta diameter; CI, confidence interval; IVC_E_, inferior vena cava diameter of end-expiratory; LVD, left ventricular diameter; LVEI, left ventricular eccentricity index; mPA, main pulmonary artery diameter; PH, pulmonary hypertension; RAA, right atrial area; RVD, right ventricular diameter; RVOT-AT, right ventricular outflow tract acceleration time; sPAP, systolic pulmonary arterial pressure; TAPSE, tricuspid annular plane systolic excursion; TRV, tricuspid regurgitation velocity; TTE, transthoracic echocardiography.

**Table S3:** Multivariate logistic regression (Enter method) including all parameters across different TR scenarios.

| Parameters | Odds Ratio | 95% CI | *p* |
| --- | --- | --- | --- |
| Excluding TR related parameters |  |  |  |
| RVOT-AT (< 93 ms) | 10.59 | 3.90-28.77 | <0.001 |
| mPA (> 25 mm) | 4.03 | 0.86-18.98 | 0.078 |
| RAA (> 14.5 cm^2^) | 2.05 | 0.62-6.82 | 0.241 |
| RVD (> 26.5 mm) | 1.44 | 0.26-7.98 | 0.676 |
| RVD/LVD (> 0.65) | 0.56 | 0.10-3.09 | 0.734 |
| mPA/AO (> 1) | 0.76 | 0.16-3.68 | 0.734 |
| LVEI (> 1.1) | 4.41 | 0.71-27.53 | 0.112 |
| IVC_E_ (> 13.5 mm) | 2.19 | 0.80-5.99 | 0.126 |
| Including TR related parameters |  |  |  |
| RVOT-AT (< 93 ms) | 7.80 | 2.50-24.33 | <0.001 |
| sPAP (> 37.5 mmHg) | 3.62 | 0.73-18.01 | 0.116 |
| TAPSE/sPAP (< 0.50 mm/mmHg) | 2.42 | 0.59-9.87 | 0.219 |
| TRV (> 2.8 m/s) | 1.23 | 0.28-5.40 | 0.787 |
| mPA (> 25 mm) | 3.06 | 0.49-19.11 | 0.231 |
| RAA (> 14.5 cm^2^) | 1.46 | 0.38-5.70 | 0.584 |
| RVD (> 26.5 mm) | 1.78 | 0.21-12.63 | 0.565 |
| RVD/LVD (> 0.65) | 0.22 | 0.03-1.63 | 0.138 |
| mPA/AO (> 1) | 0.43 | 0.07-2.76 | 0.370 |
| LVEI (> 1.1) | 14.31 | 1.05-195.66 | 0.046 |
| IVC_E_ (> 13.5 mm) | 1.57 | 0.50-4.96 | 0.443 |

AO, aorta diameter; IVC_E_, inferior vena cava diameter of end-expiratory. LVD, left ventricular diameter; LVEI, left ventricular eccentricity index; mPA, main pulmonary artery diameter; RAA, right atrial area; RVD, right ventricular diameter; RVOT-AT, right ventricular outflow tract acceleration time; sPAP, systolic pulmonary arterial pressure; TAPSE, tricuspid annular plane systolic excursion; TRV, tricuspid regurgitation velocity.

| Parameters | Inter-observer variability | | | Intra-observer variability | | |
| --- | --- | --- | --- | --- | --- | --- |
|  | ICC | 95% CI | *p* | ICC | 95% CI | *p* |
| RVD | 0.86 | 0.47-0.96 | <0.001 | 0.97 | 0.92-0.99 | <0.001 |
| LVD | 0.71 | 0.09-0.91 | 0.019 | 0.88 | 0.68-0.96 | <0.001 |
| mPA | 0.76 | 0.16-0.93 | 0.015 | 0.85 | 0.86-0.95 | <0.001 |
| RAA | 0.91 | 0.72-0.98 | <0.001 | 0.98 | 0.97-0.99 | <0.001 |
| RAP | 0.78 | -0.01-0.94 | 0.001 | 0.79 | 0.43-0.93 | 0.002 |
| TRV | 0.77 | 0.24-0.93 | 0.010 | 0.97 | 0.92-0.99 | <0.001 |
| sPAP | 0.77 | 0.26-0.93 | 0.008 | 0.98 | 0.94-0.99 | <0.001 |

**Table S4.** The inter- and intra-observer variability of certain TTE parameters determined by interclass correlation coefficients.

CI, confidence interval; ICC, interclass correlation coefficients; LVD, left ventricular diameter; mPA, main pulmonary artery diameter; RAA, right atrial area; RAP, right atrial pressure; RVD, right ventricular diameter; sPAP, systolic pulmonary arterial pressure; TRV, tricuspid regurgitation velocity.


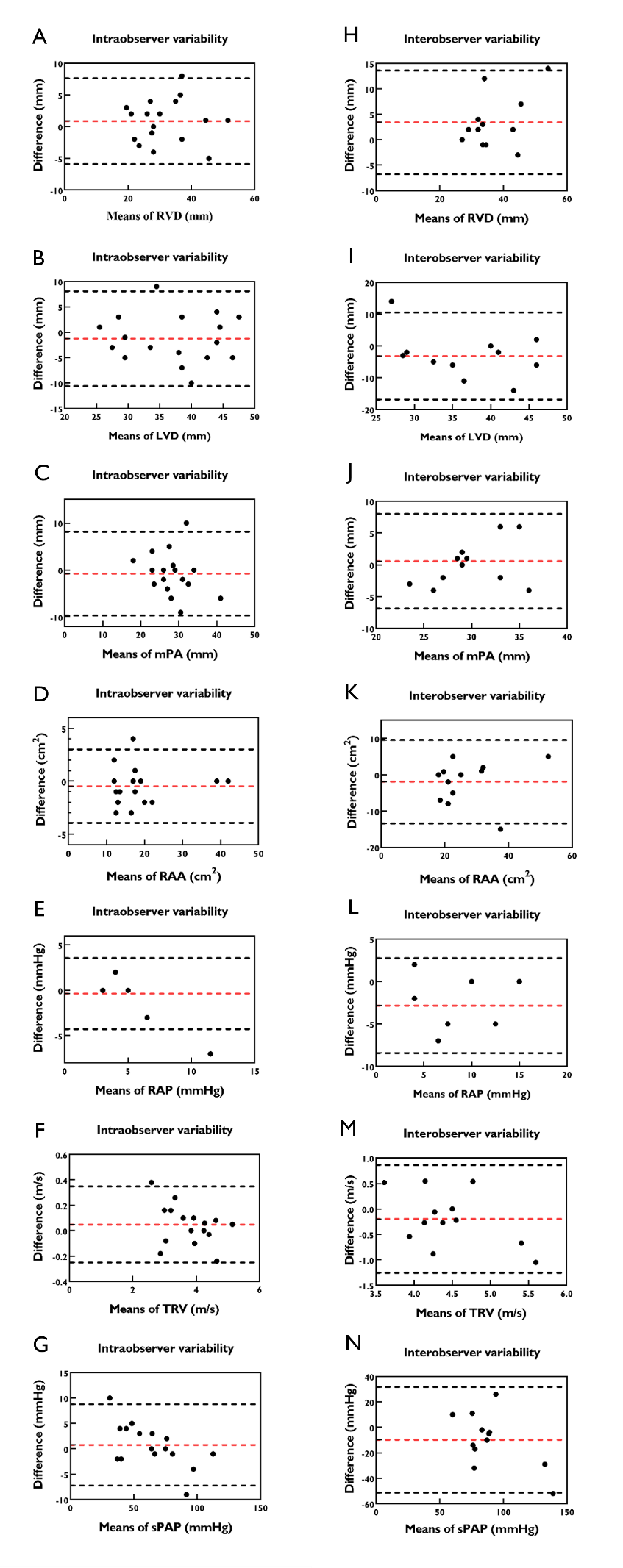


**Figure S1**. The Bland-Altman plots present intraobserver and interobserver variabilities of right ventricular diameter (RAD) (A and H), left ventricular diameter (LVD) (B and I), main pulmonary artery diameter (mPA) (C and J), right atrial area (RAA) (D and K), right atrial pressure (RAP) (E and L), tricuspid regurgitation velocity (TRV) (F and M) and systolic pulmonary artery pressure (sPAP) (G and N).
